# The Effect of Stem Cell Transplantation on Immunosuppression in Living Donor Renal Transplantation: A Clinical Trial

**Published:** 2013-11-01

**Authors:** H. L. Trivedi, A. V. Vanikar, V. B. Kute, H. V. Patel, M. R. Gumber, P. R. Shah, S. D. Dave, V. B. Trivedi

**Affiliations:** 1 *Department of Nephrology and Transplantation Medicine, *; 2 *Department of Pathology, Laboratory Medicine, Transfusion Services and Immunohematology, G. R. Doshi and K. M. Mehta Institute of Kidney Diseases and Research Centre and Dr. H. L. Trivedi Institute of Transplantation Sciences (ITS), Asarwa, Ahmedabad-380016, Gujarat, India*

**Keywords:** T-Lymphocytes, regulatory, Mesenchymal stem cells, Hematopoietic stem cells, Renal transplantation, Clonal deletion, Immunosuppression, Graft rejection

## Abstract

Background/Objective**:** We designed a clinical trial on a group of live-donor renal transplantation (LDRT) patients subjected to pre-transplant stem cell transplantation (SCT) to minimize immunosuppression to low-dose steroid monotherapy.

Methods**:** LDRT patients subjected to pretransplant SCT who had stable graft function for ≥2 years and serum creatinine (SCr) <2 mg/dL were recruited. Patients with diabetes, hepatitis C/B, rejections, or unwilling to participate, were excluded. They had been subjected to non-myeloablative conditioning of total lymphoid irradiation (TLI)/bortezomib and cyclophosphamide, rabbit-antithymocyte globulin (r-ATG) and rituximab with SCT. The maintenance immunosuppression consisted of calcineurin inhibitors (CNI) and/or anti-proliferative agents and prednisone. Donor-specific antibodies (DSA) and peripheral T-regulatory cells (CD127^low/–^/4^+^/25^high^) (p-Tregs) were studied before and after withdrawal of major immunosuppressants; graft biopsy was taken after 100 days of withdrawal in willing patients. Rejections were planned to be treated by anti-rejection therapy followed by rescue immunosuppression.

Results**:** All immunosuppression but prednisone, 5–10 mg/day has been successfully withdrawn for a mean of 2.2 years in 76 patients with a mean age of 31.4 years and a mean donor-recipient HLA match of 2.9. The mean SCr of 1.4 mg/dL and p-Tregs of 3.5% was remained stable after withdrawal; DSA status was negative in 35.5% and positive in 47.4% patients. Protocol biopsies in all 10 patients who gave the consent were unremarkable.

Conclusion**:** Stable graft function in LDRT on low-dose steroid monotherapy using pre-transplant SCT under non-myeloablative conditioning with generation of p-Tregs can be achieved successfully and safely.

## INTRODUCTION

Kidney transplantation developed as a surgical art rather than an immunological intervention when the first deceased-donor kidney transplantation was performed in the USA in 1950 and lasted for 10 months. The first live donor renal transplantation (LDRT) was performed in 1954 in Boston and Paris [1]. However, there was little knowledge of the underlying immune mechanisms and no surprise, no immunosuppressive therapy was available then.

With advancement in the field of transplantation, surgical skills have developed extensively; however, immunological mechanisms involved in the process of rejection of a transplant were still ill-understood. Immunosuppressants/deletional agents have been able to control acute rejections; nonetheless, the problem of chronic rejections remained unanswered.

Previously stem cells (SC) therapy was used to minimize the level of immunosuppression necessary for transplantation [2, 3]. Here, we conducted this clinical trial to determine if we can minimize the level of immunosuppression to monotherapy with prednisone, 5-10 mg/day in a cohort of willing LDRT patients who had undergone pre-transplant SC therapy. We studied the donor-specific antibodies (DSA) and peripheral T-regulatory cells (CD127^low/–^/CD4^+^/CD25^high^) (p-Tregs) along with serum creatinine (Cr).

## PATIENTS AND METHODS

Seventy-six (68 male, 8 female) patients with mean±SD age of 31.4±10.2 years and donor-recipient HLA match of 2.92±1.34 were subjected to immunosuppression minimization. There were 12 patients with no DR match, 50 patients with 1 DR match and 14 with full DR match. Donors were parents in 46 patients, spouses in 15, siblings in 11, son in one and unrelated in three patients. The underlying diseases were chronic glomerulonephritis in 44 patients, reflux nephropathy in eight, hypertensive nephropathy in seven, chronic tubulointerstitial nephritis in six, obstructive uropathy in three, vasculitis in two, Alport’s syndrome in two, and autosomal dominant polycystic kidney disease, focal segmental glomerulosclerosis, membranous nephropathy, and lupus nephritis, one each.

All these patients underwent LDRT using pre-transplant SC therapy between September 1998 and August 2010. Inclusion criteria were stable graft function for ≥2 years with Cr <2.0 mg/dL and absence of rejections. Unwilling patients, diabetics and those with hepatitis C/B were excluded from the trial. 

All patients received donor hematopoietic stem cells (HSCT) (mean±SD: 17.4±5.5 × 10^8^ nucleated cells/ kg BW of recipient with mean±SD CD34^+^ count of 2±0.7 × 10^4^/kg BW). In addition, donor adipose-tissue-derived mesenchymal stem cells (AD-MSC) (with mean±SD CD45^–^/90^+^ cells of 5.8±1.5 × 10^4^/ kg BW and CD45^–^/73^+^ cells of 1.1±0.92 ×10^4^/kg BW) were infused in 55 patients. Non-myeloablative conditioning of cyclophosphamide, 20 mg/kg BW, rabbit-antithymocyte globulin (r-ATG), 1.5 mg/kg BW and rituximab, 375 mg/m^2^ was given to all patients. In addition, 41 patients had undergone total lymphoid irradiation (TLI), (200 cGy × 5 times) as part of conditioning, and 25 patients received bortezomib, 1.3 mg/m^2^ × 4 times along with methylprednisone, 125 mg intravenously instead of TLI prior to transplantation. DSA (Luminex single antigen assay) and p-Tregs (flowcytometry) were tested before minimization of immunosuppression and three months after withdrawal of the principal immunosuppressants. 

DSA measurement 

Class I and II antibody specificity screening was performed with single antigen beads (One Lambda, Canoga Park, CA, USA). Screening tests for anti-HLA-specific IgG antibodies was performed using LABScreen^®^ single antigen beads, class I and II (One Lambda Inc., Canoga Park, CA, USA). The assays were performed on Luminex platform following the manufacturer’s protocol. Trimmed mean fluorescence intensity (MFI) values were obtained from the output file generated by the flow-analyzer, and normalized using the formula:

(Sample #N beads – Sample negative control (NC) beads) – (Negative control serum #N beads – Negative control serum NC beads)] 

A normalized MFI value over 2000 was considered positive.

Measurement of pTregs 

pTregs (127^low/–^/CD4^+^/25^high^) was measured in peripheral blood of patients of all groups using CD127 mAb (PerCP-Cy), CD4 mAb (phycoerythrin [PE]), and CD25 mAb (fluorescein isothiocyanate [FITC]) (Becton Dickinson [BD] Biosciences) according to manufacturer’s protocol using FACScan (BD Biosciences, USA). 

Procurement of peripheral blood stem cells (PBSCs) 

PBSCs were collected from cytokine-stimulated donors for two days. They were subjected to leucopheresis on SC separator (Cobe Spectra version 7-Gambro, China). GCSF, 300 µg, twice a day for two days, was used as cytokine for bone marrow (BM) stimulation and mobilization before procurement.

BM aspiration and processing procedure 

A total of 100 mL BM was aspirated from the posterior superior iliac crest of donors under local anesthesia and sedation (if donor was apprehensive) after the cytokine stimulation for two days (as mentioned above). 

The marrow was collected in transfer medium and transferred for culturing to SC lab to increase the yield of CD34^+ ^cells by *in vitro* expansion and fortifying un-fractionated BM with stromal cells. 

Portal infusion of SCs

Under general anesthesia, a midline incision of approximately 3–5 cm length was made above the umbilicus, omental vein was identified and canulated with a 20 G intracath. SC bag was connected and infused directly without using any filters, at a rate of 6–8 mL/min. After infusion, the omental vein was ligated with a silk suture and hemostasis was checked. The wound was closed with vicryl 2/0; subcuticular stitches were inserted using 3/0 monocryl. 

Stem cell lab protocols 

*In vitro expansion of HSC*

BM collected from donors was transferred in a self-designed medium composed of Dulbecco’s modified Eagle’s medium (DMEM) with antibiotics and then immediately shifted to culture medium composed of DMEM with high glucose, essential amino acids, albumin, growth factors and antibiotics. Medium was replenished every other day for 8–10 days, the supernatant was removed on the 7^th^/8^th^ day; the cultured marrow was mixed with AD-MSC after testing for viability, sterility, staining and quantification. 

AD-MSC

Adipose tissue was resected from the anterior abdominal wall of kidney donor under local anesthesia after making a small incision on the left lateral side below the umbilicus. Sutures were inserted after hemostasis was secured. This adipose tissue collected in a self-designed medium containing α-MEM, 20% human albumin, and antibiotics; it was taken to the lab and minced with knife into tiny pieces. Then, it was transferred in to the above medium with addition of collagenase type I, incubated at 37 °C for one hour on a self-designed shaker at 35-40 RPM for digestion. The contents of the medium processed in Petri dish, were transferred to centrifuge tubes, centrifuged at 780 RPM for eight minutes. The supernatant and pellets were separately cultured in the medium with same composition on 100 cm^2^ and 25 cm^2^ cell+ culture dishes (Sarsted, USA), respectively, at 37 °C with 5% CO_2_ for 8–10 days. The medium was replenished every other day and then harvested by trypsinization. The collected cells after being tested were subjected to flowcytometric analysis. Cells were mixed with cultured BM and infused in portal circulation of patients. 

Cell counts, viability and sterility 

The total nucleated cell counts, viability and sterility tests were performed by standard lab techniques. SCs were analyzed using FACScan (Becton Dickinson, USA). CD34^+/–^CD45^+^/CD33^+/– ^cell lines were counted. We used CD33 mAb (PE-conjugated), CD34 mAb (FITC-conjugated) and CD45 mAb (PerCP-conjugated) (BD Biosciences, USA). For AD-MSC, CD 45^–^/90^+ ^and CD73^+^, CD^73 ^mAb (PE-conjugated), CD90 mAb (FITC-conjugated) and CD45 mAb (PerCP-conjugated) were used.

Peripheral blood and BM samples were collected in EDTA. In FACS tubes 20 µL of appropriate antibody was taken and 100 µL of blood was added. After vortexing for 5 sec, the tubes were incubated in dark for 30 min. Then 2 mL of 10× lysing solution was added followed by centrifuging at 1000 RPM for 5 min. Supernatant was discarded and 2 mL of sheath fluid was added. The tubes were again centrifuged at 1000 RPM × 5 min. Supernatant was discarded. Finally, 500 µL of sheath fluid was added for blood/H-AD-MSC samples and 1 mL was added for BM samples and subjected to data acquisition. Unstained blood samples were used as negative controls.

The initial maintenance immunosuppression consisted of calcineurin inhibitor (CNI) (cyclosporin, 3 mg/kg BW/day; tacrolimus, 0.08 mg/kg BW/day) or sirolimus, 1–2 mg/day and/or mycofenolate sodium, 360 mg BD; azathioprine, 50–100 mg/day, and prednisone, 5–10 mg/day. 

CNI levels and sirolimus were measured at weekly intervals for the first two months, fortnightly for the next two months and subsequently, whenever indicated clinically, using Siemens reagent flex kit (Siemens RxL Max) according to manufacturer’s protocol with the aim of maintaining trough levels of cyclosporin between 100 and 150 ng/mL and that of tacrolimus and sirolimus between 4 and 7 ng/mL. We did not measure mycofenolate level. Immunosuppression withdrawal was started with CNI followed by anti-proliferative agents. Prednisone was continued. Rejections were planned to be treated by anti-rejection therapy followed by rescue immunosuppression. Protocol biopsies were planned after 100 days of steroid monotherapy whenever patients gave their written informed consent. Trials were approved by the Institutional Review Board.

## RESULTS

**Figure 1:**
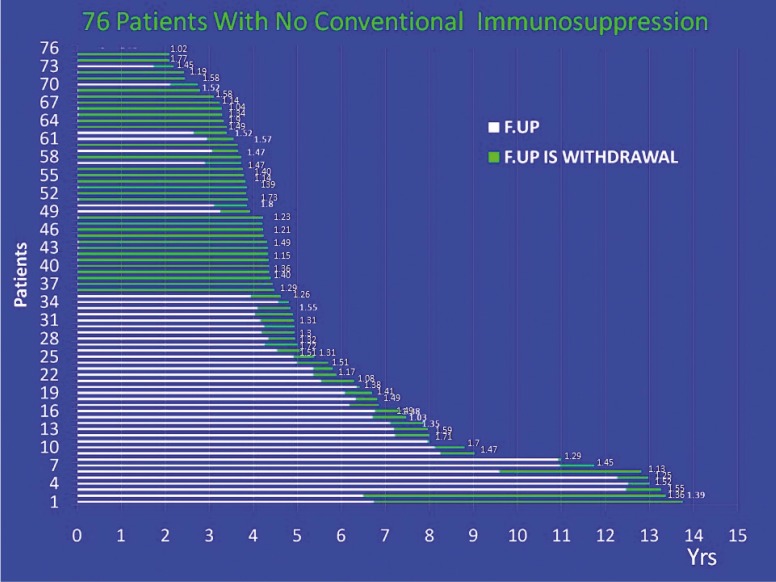
Bar diagram depicting 76 patients since they were transplanted, white part of bars shows post-transplant follow-up, green areas on bars show follow-up since immunosuppression withdrawal. At the end of each bar is their present serum creatinine level (mg/dL). X axis is the time in years post-transplantation

**Figure 2:**
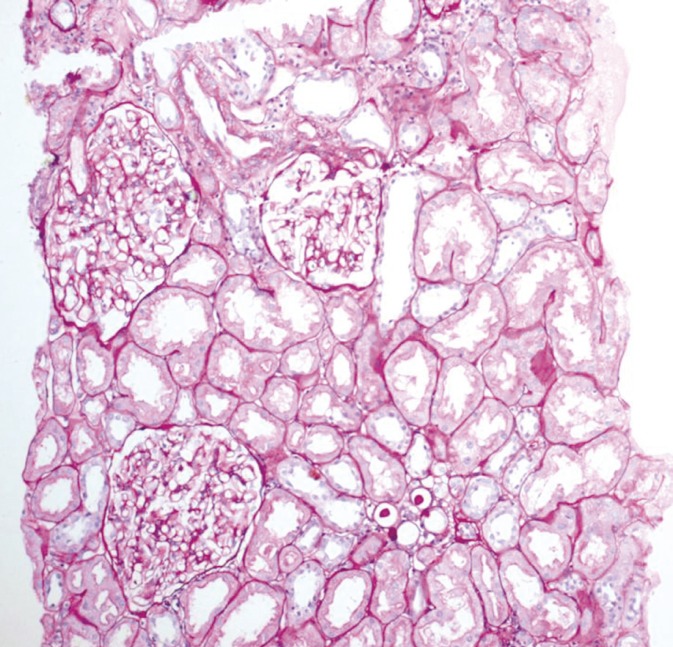
Protocol biopsy of a 47-year-old woman performed on June 27, 2012, transplanted with father-in-law’s kidney (HLA 0/6 match) on July 18, 2003. Histopathology demonstrating normal renal allograft morphology with three glomeruli, one medium artery and surrounding tubules. PAS stain, 100×.

All immunosuppression but prednisone has been successfully withdrawn in all 76 patients (Fig 1). No adverse events/rejections were recorded after withdrawal. The mean±SD post-transplant follow-up was 5.5±3 years; the mean±SD follow-up since steroid monotherapy was 2.2±1.8 years. DSA-class-1 were present in 15 (20%), class-2 in 11 (15%), and both in 10 (13%) patients; none of them were exist in 27 (36%) patients. The same status remained even with steroid monotherapy. Thirteen (17%) patients are still to be tested. Protocol biopsies performed in all 10 patients who gave the consent, were unremarkable (Fig 2). Out of the biopsied patients, four had no antibodies, four had HLA class-1, and two had HLA class-2 DSA. The mean±SD p-Tregs was 3.53%±1.35%; the mean Cr level was 1.4±0.2 mg/dL at the time of immunosuppression withdrawal and remained at that level thereafter. 

## DISCUSSION

Organ transplantation has become a well-accepted therapeutic modality since the first successful LDRT performed in 1954 following successful skin transplantation in rodent model [1,4]. Survival of the transplants and patients depends on many factors—immunological and non-immunological. The principal pathways of rejection are direct and indirect, which involve the antigen presentation by antigen-presenting cells from both the donor and recipient, in addition to interplay of T-cells and B-cells with involvement of HLA antigens. 

Medawar’s school of thought believed that abrogation of T-cell responses by central and peripheral tolerance would lead to induction of tolerance. This would involve chimeric tolerance, anergy and blockade of co-stimulatory pathway [5-8]. Gorer’s school was a firm believer of B-cell pathway and that overcoming the hurdle of antibody-mediated rejection would lead to the promised land of tolerance [9-11]. 

With the understanding of these concepts, anecdotal reports have been published on tolerance induction, however, no definite reproducible model of deliberately induced and sustained clinical tolerance has evolved [12-23]. Our journey to the promised land of tolerance began in 1998 with megadose of HSC.2 We kept on modifying our tolerance induction protocols with improvement in our understanding [24]. 

The reason for using megadose of HSC using PBSCs was that they are chiefly composed of donor T-cells along with a small dose of CD34^+^ SC [25, 26]. Double negative (CD3– CD4– CD8– ) putative T-regs in PBSCs may counteract anti-donor T cells, both systematically and locally, as well as infiltrate the graft, thereby facilitating graft and donor cell survival [27, 28]. Portal infusion of SC was planned because liver is the most tolerogenic organ. It helps in achieving prope’ tolerance with low-grade lymphohematopoietic chimerism [29, 30]. We made major changes subsequently by reducing the PBSC dose, adding donor-specific transfusions (DST) and aspirating donor BM for infusion into the portal peripheral circulation, as well as the marrow and thymus. This study was implemented in the belief that classical central tolerance can only be achieved by directly injecting SCs into the thymus, which preserving substantial numbers of T-regs in the recipient lymphoid repertoire as well as seeking apoptosis of activated T cells [31, 32]. We added non-myeloablative conditioning treatment to create space in the marrow and reticuloendothelial systems, using cyclophosphamide and rabbit antithymocyte globulin to delete stimulated T-cell clones and rituximab to delete stimulated B-cell clones and control antibody response [33]. However, our patients still required conventional immunosuppression. Therefore, in the fourth protocol we modified the regimen by adding another DST as well as target-specific irradiation (TSI) [34]. Radio-resistant NKT cells (natural killer T-cells) were thus able to interact with antigen-presenting cells. However, we omitted thymic inoculation, which was deemed not acceptable. In the fifth protocol, we converted TSI to total lymphoid irradiation (TLI) and omitted intra-marrow and intra-thymic infusion of BM [34]. At this stage, we began to culture BM to generate a larger yield of CD34^+^ SC. In the sixth protocol, we stimulated major histocompatibility complex (MHC)-restricted T- and B-cell clones and then deleted them. We used donor adipose tissue-derived mesenchymal SCs (ad-MSC) with PBSC and HSCT, omitting DST and TLI [35, 36]. We used the proteasome inhibitor bortezomib to delete plasma cells [37, 38]. From this protocol onward, we employed T- and B-cell flow crossmatches. Eventually, we realized that we have developed a clinical model of LDRT on low-dose steroid monotherapy using pre-transplant SC therapy under non-myeloablative conditioning with generation of p-Tregs. We believe that p-Tregs protect these grafts from chronic rejections. SC, especially MSCs exhibit their genetically unrestricted immunosuppressive effects by inhibition of proliferation and function of T-cells, B-cells and NK cells in a dose-dependent manner. MSCs also have tolerogenic effect by which they prolong survival of organ grafts and prevent graft *vs* host disease. MSCs avoid allogenic rejection by being hypoimmunogenic, modulating T-cell phenotype and by creating an immunosuppressive local milieu. Thus, MSC exhibit immunogenicity, “tolerogenicity,” and immunosuppressive effects [25-30, 39-43]. Control of chronic rejection, which occurs through the indirect pathway, has been achieved in our model with sustained presence of p-Tregs. We believe that we have generated p-Tregs from SC therapy [31, 32, 44, 45]. 

This study shows promising clinical results in achieving successful minimization of immunosuppression in LDRT to low-dose steroid monotherapy. In this study, we did not carry out chimerism studies because our previous experience indicated that peripheral blood chimerism may not be associated with absence of rejection episodes and *vice versa* [3, 33]. Protocol biopsies were performed in a limited number of patients because it is not easy to obtain consent from patients. However, we are trying to perform protocol biopsies in more patients. Financial constraints were the major shortcomings for performing more frequent immunological monitoring. However, we have already started performing DSA at three monthly intervals. Multi-center trials will prove the beneficial effects mentioned here. However, the major problem could be in replicating the *in vitro* generation of adipose tissue derived MSC and a protocol which may require longer hospital stay.

In conclusion, we have achieved successful minimization of immunosuppression to low-dose steroid monotherapy in LDRT using pre-transplant SC therapy. We have also observed that p-Tregs (CD127^low/–^/CD4^+^/CD25^high^) are generated in this process.

## Notes

**FINANCIAL SUPPORT:** None.
